# Genome-wide methylome profiling of cell-free DNA enables prognostication of patients with castration-resistant prostate cancer

**DOI:** 10.1038/s41416-026-03432-y

**Published:** 2026-04-10

**Authors:** Karoline Kondrup, Laura Iisager, Paul Vinu Salachan, Maibritt Nørgaard, Philippe Lamy, Rosalind Eeles, Torben Frøstrup Hansen, Palle Jörn Sloth Osther, Ahmed Hussein Zedan, Michael Borre, Karina Dalsgaard Sørensen

**Affiliations:** 1 Department of Molecular Medicine, Aarhus University Hospital, Aarhus, Denmark; 2 Department of Clinical Medicine, Aarhus University, Aarhus, Denmark; 3 The Institute of Cancer Research, London, UK; 4 Royal Marsden NHS Foundation Trust, London, UK; 5 Department of Regional Health Research, University of Southern Denmark, Odense, Denmark; 6 Department of Oncology, Lillebaelt Hospital, University Hospital of Southern Denmark, Vejle, Denmark; 7 Department of Urology, Vejle Hospital, University Hospital of Southern Denmark, Vejle, Denmark; 8 Department of Urology, Aarhus University Hospital, Aarhus, Denmark; 9 Present Address: ARCEDI, Aarhus, Denmark

**Keywords:** Prognostic markers, Prostate cancer, Tumour biomarkers

## Abstract

**Background:**

Metastatic castration-resistant prostate cancer (mCRPC) remains a lethal disease with few biomarkers to inform treatment selection or patient prognosis. Methylation profiles of plasma circulating tumour DNA (ctDNA) accurately reflect tumour methylomes and may reveal novel biomarkers of mCRPC.

**Methods:**

To establish a novel mCRPC-associated methylation signature for detection of ctDNA, we performed plasma methylome profiling on 27 mCRPC patients and 10 controls (cohort 1). Signature-based ctDNA detection was evaluated across prostate cancer (PC) disease stages using an internal cohort of 93 PC patients and 8 controls (cohort 2), and an external cohort of 115 PC patients (cohort 3).

**Results:**

We established a 48-region methylation signature (cfMeCaP) capable of highly sensitive detection of ctDNA in mCRPC (100%, 84% and 95% in cohorts 1, 2 and 3, respectively). cfMeCaP methylation at mCRPC diagnosis was associated with poor progression-free survival (PFS) and overall survival in all three cohorts (*p* < 0.005), independent of routine clinical variables. Persistent serial detection of ctDNA using cfMeCaP was strongly associated with rapid mCRPC treatment failure (median PFS 4.4 vs. 65.5 months; *p* < 0.0001), while no detection predicted continued treatment response.

**Conclusion:**

These results highlight cfMeCaP as a promising non-invasive biomarker for prognostication in mCRPC.

## Background

Metastatic castration resistant prostate cancer (mCRPC) presents a significant healthcare challenge worldwide due to high mortality rates [1, 2]. Standard treatment options, such as taxanes and androgen receptor signalling inhibitors (ARSI), provide only limited survival benefits owing to high levels of primary and acquired resistance [3, 4]. Currently, no method can accurately predict treatment resistance in mCRPC and routine tools to monitor therapy efficacy, i.e. prostate specific antigen (PSA) and imaging, remain suboptimal [3, 5]. Moreover, tumour tissue-based molecular analyses are challenging in this setting, as metastases are not routinely sampled. Hence, there is an urgent need for novel, minimally invasive biomarkers to guide personalised treatment. Plasma cell-free DNA (cfDNA) offers a promising strategy for biomarker discovery in mCRPC patients as a fraction of cfDNA may be tumour cell-derived (circulating tumour DNA; ctDNA) and has been shown to reflect the molecular alterations of matched tumour tissue [6].

Here, we employed a highly sensitive methodology, cell-free methylated DNA immunoprecipitation and high-throughput sequencing (cfMeDIP-seq) [7, 8], to characterise the methylome landscape of mCRPC using plasma cfDNA. By cfMeDIP-seq methylome profiling of plasma cfDNA samples from a cohort of 27 patients with mCRPC and 10 men without PC, we aimed to develop a mCRPC-associated cfDNA methylation signature and subsequently evaluate the signature in large independent cohorts, comprising more than 700 samples from 388 prostate cancer (PC) patients and 305 male controls. We established a novel 48-region mCRPC-associated cfDNA methylation signature (cfMeCaP) with high sensitivity and specificity for detection of methylated ctDNA (me-ctDNA) in plasma. We found high cfMeCaP methylation levels at mCRPC diagnosis to be associated with poor PSA progression-free survival (PSA-PFS) and poor overall survival (OS). Furthermore, during first-line ARSI treatment, longitudinal me-ctDNA detection analyses based on cfMeCaP could identify patients with a high risk of early treatment failure.

## Materials and methods

### Cohorts for cfMeDIP-seq analysis

Cohort 1 comprised of 27 patients with mCRPC (mCRPC) and 10 men without PC (non-PC control; NPCC), recruited at the Department of Urology, Aarhus University Hospital (AUH), Denmark (2016-2020).

Cohort 2 comprised of 43 patients with mCRPC (mCRPC), 40 with hormone sensitive PC (HSPC), 10 with localised PC (LPC) and 8 men without PC (NPCC), recruited at the Department of Urology, AUH (*n* = 79; 2006–2022) or at the Department of Oncology, Vejle Hospital, Denmark (*n* = 22; 2016–2020).

In both cohorts, metastases were verified by CT scan, bone scintigraphy or ^18^F-sodium fluoride PET (F-18Na/PET). Blood samples from all mCRPC patients (cohort 1 and cohort 2) were drawn immediately before the initiation of first-line mCRPC treatment (enzalutamide or abiraterone). LPC blood samples were drawn at the time of radical prostatectomy (RP). HSPC blood samples were collected immediately before initiating androgen deprivation therapy (ADT; *n* = 37) or during ADT (*n* = 3). All NPCC men had low serum PSA levels (<4 ng/ml), and prostate biopsies confirmed to be negative for PC. Clinical characteristics for both cohorts are shown in Table 1.

**Table 1:** Patient characteristics.

	Cohort 1		Cohort 2			
	NPCC (*n* = 10)	mCRPC (*n *= 27)	NPCC (*n* = 8)	LPC (*n* = 10)	HSPC (*n* = 40)	mCRPC (*n* = 43)
Age (year)						
Median	66	72	71	64	75	74
(Range)	(42–83)	(60-87)	(54–85)	(61–70)	(58–87)	(42–86)
Serum PSA level (ng/mL)						
Median	1.3	108.0	0.9	13.7	23.4	43.8
(Range)	(0.6–3.9)	(13.2–759.4)	(0.3–14.5)	(5.5–37.0)	(0.4–318.0)	(3.0–288.0)
Plasma cfDNA concentration (ng/mL)						
Median	4.7	28.7	6.4	4.5	7.7	7.5
(Range)	(4.1–15.1)	(7.5–165.0)	(3.6–11.5)	(3.0–10.1)	(3.4–26.5)	(4.1–21.4)
Metastatic tumour burden at sample collection: *n* (%)						
M0	-	-	-	10 (100.0)	6 (15.0)	-
M1 - Bone only	-	9 (33.3)	-	-	10 (25.0)	17 (39.5)
M1 - Lymph node only	-	2 (7.4)	-	-	10 (25.0)	5 (11.6)
M1 - Bone and lymph node	-	11 (40.7)	-	-	12 (30.0)	17 (39.5)
M1 - Visceral	-	5 (18.5)	-	-	-	2 (4.7)
M1 - Unknown	-	-	-	-	2 (5.0)	2 (4.7)
Therapy before sample collection, *n* (%)						
No prior treatment	-	-	-	10 (100.0)	29 (72.5)	-
Radical prostatectomy	-	2 (7.4)	-	-	3 (7.5)	3 (7.0)
Radiotherapy	-	4 (14.8)	-	-	6 (15.0)	5 (11.6)
Antiandrogen blockage	-	9 (33.3)	-	-	11 (27.5)	10 (23.3)
ADT (GnRH/LH-RH-agonist)	-	27 (100.0)	-	-	3 (7.5)	39 (90.7)
Surgical castration	-	-	-	-	-	4 (9.3)
Taxanes (docetaxel/cabazitaxel)	-	9 (33.3)	-	-	1 (2.5)	9 (20.9)
mCRPC 1st-line treatment, *n* (%):						
Enzalutamide	-	24 (88.9)	-	-	-	37 (86.0)
Abiraterone	-	3 (11.1)	-	-	-	6 (14.0)
PSA progression, 1st-line mCRPC treatment						
Yes, *n* (%)	-	24 (88.9)	-	-	-	30 (69.8)
PSA progression-free survival (months), median (range)	-	5.3 (1.3–32.4)	-	-	-	9.8 (1.7–65.5)
No, *n* (%)	-	3 (11.1)	-	-	-	13 (30.2)
Available follow-up time (months), median (range)	-	17.7 (3.8–53.0)	-	-	-	34.5 (2.9–88.4)
Dead						
Yes, *n* (%)	-	19 (70.4)	-	-	-	19 (55.8)
Overall survival Months, median (range)	-	13.1 (3.6–38.6)	-	-	-	18.6 (5.2–35.3)
No, *n* (%)	-	8 (29.6)	-	-	-	24 (44.2)
Available follow-up time Months, median (range)	-	13.6 (2.6–56.2)	-	-	-	37.6 (2.9–88.4)

Clinical characteristics of non-prostate cancer controls (NPCCs) and prostate cancer (PC) patients in cohorts 1 and 2. *mCRPC* metastatic castration-resistant PC, *LPC* localised PC, *HSPC* hormone-sensitive PC, *PSA* prostate specific antigen, *ADT* androgen deprivation therapy.

Cohort 3 (external validation) comprised 30 patients with localised PC (LPC) and 85 patients with mCRPC (mCRPC) recruited in USA and Canada [9]. Samples were collected at the time of RP (LPC patients) or at mCRPC baseline before initiation of first-line enzalutamide or abiraterone treatment (mCRPC patients). We downloaded cfMeDIP-seq data for patients in this cohort from EGAS00001005522. Additionally, we downloaded clinical follow-up data available for 72 out of the 85 mCRPC patients in this cohort [9, 10]. Clinical characteristics are shown in Table S1.

### Methylation array data

We downloaded Infinium 450 K methylation array data from the Marmal-aid database [11] for 1057 blood cell samples, collected from men without cancer and of varying ages and ethnicities (referred to as Marmal-aid blood cell (MBC) dataset), as well as for 180 primary prostate tumour and 52 healthy prostate tissue samples (referred to as Marmal-aid tissue (MAT) dataset). Furthermore, we downloaded Infinium Methylation EPIC BeadChip data of blood cell samples from 245 healthy men (referred to as EPIC dataset) from GSE152026 [12]. Further details are available in the Supplementary Methods and Table S2.

### Sample processing and library preparation

The blood samples from patients in cohorts 1 and 2 were processed according to standard protocols [13], with plasma separated from cellular components by centrifugation (3000 × *g* for 10 min at 20 °C) within 2 h of blood draw. cfDNA was extracted from 2 to 4 mL of plasma using the QIAamp Circulating Nucleic acid kit (Qiagen) following the manufacturer’s instructions. cfDNA concentration and quality were evaluated by multiplex droplet digital PCR (ddPCR), as described previously [13], using the QX200 AutoDG ddPCR System (Bio-Rad). Samples were split into immunoprecipitated (IP) for cfMeDIP-seq and input control (IC) for low-pass whole genome sequencing (LPWGS). Libraries were prepared in accordance with the previously published protocol [7], with slight adjustments in reaction volumes to accommodate a lower input cfDNA concentration. Further details are provided in the Supplementary Methods.

### Sequencing and initial data processing

Indexed cfMeDIP-seq libraries were paired-end sequenced (2 × 150bp) on an Illumina® Novaseq 6000 instrument (S-prime, S1, or S2 flow cell). Sequencing generated a median of 57.9 (14.3–113) million reads per IP library and 19.6 (3.8–68.5) million reads per IC library, corresponding to median coverages of 1.47x (0.27–4.27x) for IP libraries and 0.53x (0.08–3.37x) for IC libraries (Table S3). Raw Fastq files were demultiplexed (bcl2fastq, v2.20.0.422), and adaptor sequences were trimmed (Cutadapt, v1.16). Sequence reads were aligned and mapped to the hg19 reference genome (BWA MEM, v0.7.15), and PCR and optical duplicates were removed from each library (Samblaster, v0.1.24). The final bam files were realigned (GATK, v3.8.1.0), and Picard tools (v2.0.1) were used to calculate basic alignment statistics.

### Bioinformatics processing of sequencing data

IC sample bam files were used as input for ichorCNA [14] to produce copy number profiles (bin size=10 kb) and estimate ctDNA fraction (ctDNA%). Samples with a ctDNA fraction above 3.0% were considered as ctDNA positive (Table S3). In silico quality control (QC) was evaluated for each IP and IC sample using the MEDIPS R package (v1.42.0) [15]. All samples passed initial QC, as they demonstrated efficient capture of methylated cfDNA fragments (median 2.8-fold enrichment in IP relative to IC samples) as well as sufficient sequencing depth (median depth of 1.47x for IP and 0.53x for IC samples; Table S3). Whole-methylome profiles were generated from IP sample bam files using the QSEA R package (v1.16.0) [16]. For each sample, read coverage was estimated per 300 base pair (bp) window across the genome. For normalisation of coverage data, copy number profiles of corresponding IC samples were imported into the QSEA workflow. For analysis, coverage per 300 bp window was converted into normalised reads per million mapped reads (nrpm).

### Methylome landscape analysis

Differential methylation between mCRPC and NPCC samples in cohort 1 was analysed using QSEA, and 300 bp windows with a false-discovery rate (FDR) corrected *p*-value < 0.05, and absolute log_2_ fold change (log_2_FC) > 1.5 (hyper) or <−1.5 (hypo) were nominated as differentially methylated regions (DMRs). The genome-wide distribution of DMRs was visualised using the RIdeogram R package (v0.2.2). DMRs were annotated in relation to CpG and genic annotations using Bioconductor’s Annotatr package (v1.16.0) [17].

### mCRPC signature creation using methylation data

We used DMRs identified in the methylome landscape analysis for the mCRPC signature creation. To improve the PC-specificity of the DMRs, we first filtered the DMRs using the MBC data to contain only DMRs that were commonly unmethylated in male blood cells, defined as: mean beta of the DMR < 0.05, and <1 × standard deviation.

We then used the cohort 1 methylation values to select DMRs that were highly hypermethylated in mCRPC patients (>1.2 nrpm), while showing very low methylation signal in NPCC (<0.05 nrpm). The resulting DMRs formed the methylation signature, with mean methylation levels (nrpm) of this signature reported hereafter.

For detection of me-ctDNA using the methylation signature, a fixed cutoff (me-ctDNA detection cutoff) was set based on cohort 1 NPCC, as: mean signature methylation (NPCC) + 2 × standard deviation (NPCC).

Me-ctDNA detection in a sample was then defined as: mean signature methylation (sample) > me-ctDNA detection cutoff.

### Longitudinal me-ctDNA analyses during 1st-line mCRPC treatment

The changes in me-ctDNA during enzalutamide treatment were assessed using longitudinally collected plasma samples (at weeks 12 and 24) available for 22 mCRPC patients in cohort 2 (see Supplementary Methods). Based on the longitudinal me-ctDNA detection status, patients were divided into 2 groups: (1) Persistent: me-ctDNA detectable at baseline and in at least one of the two follow-up samples, (2) Non-persistent: me-ctDNA undetectable at baseline OR detectable at baseline but undetectable in both follow-up samples. Me-ctDNA dynamics during treatment were then evaluated in association to the duration of therapy response, assessed by serum PSA measurements and routine scans (bone scintigraphy or F-18Na/PET), respectively.

### Statistical data analysis

All statistical analyses were conducted in R (v4.0.2), with two-sided *p*-values < 0.05 considered statistically significant. When appropriate, *p*-values were adjusted to correct for multiple testing using the Benjamini-Hochberg method. Mann-Whitney, Kruskal-Wallis, and Spearman’s correlation tests were used to compare groups. Exact McNemar’s test was used to compare sensitivity between ctDNA detection strategies. Code for analyses conducted in R is publicly available on GitHub at https://github.com/kkt8000/cfMeCaP-code.

Survival analyses, including Kaplan-Meier analyses and uni- and multivariate Cox regression analyses, were performed using the survminer (v0.4.9), survival (v3.1-12), and forestmodel (v0.6.2) R packages. PSA-PFS and OS from the time of treatment initiation were used as endpoints for survival analyses. Signature methylation levels were either included in the analyses as continuous variables, or patients were dichotomised into two groups (low and high signature methylation) based on the median methylation level of the specific cohort. For cox regression analyses, variables violating the proportional hazards assumptions were excluded. Further details are available in the supplementary methods.

## Results

### The cfDNA methylome landscape of mCRPC investigated by cfMeDIP-seq

**Fig. 1:**
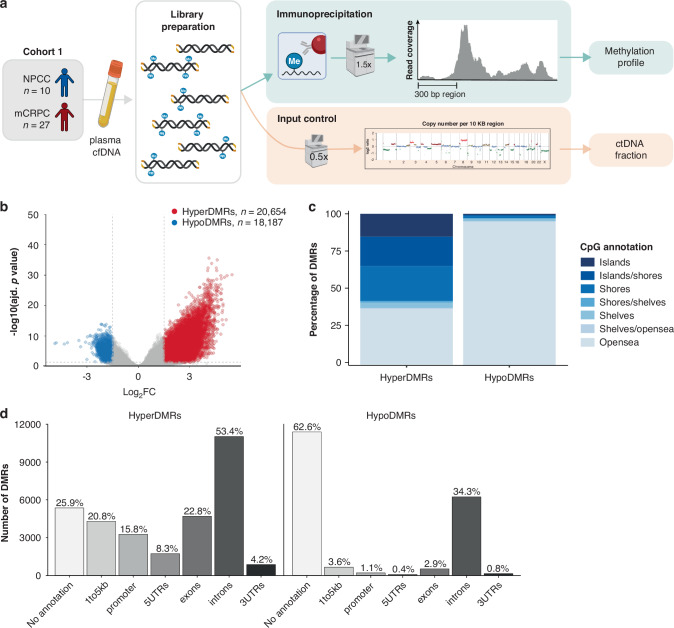
Whole-methylome profiling of mCRPC cell-free DNA. **a** cfMeDIP-seq workflow, including generation of whole-genome methylation profiles from immunoprecipitated (IP) samples and copy-number profiles from input control (IC) samples. **b** DMR analyses of cohort 1. Red dots represent regions being hypermethylated in mCRPC patients relative to NPCCs (hyperDMRs: adj. *p* < 0.05, log fold change > 1.5). Blue dots represent regions being hypomethylated in mCRPC patients relative to NPCCs (hypoDMRs: adj. *p* < 0.05, log fold change < −1.5). **c** CpG annotation of cohort 1 hyperDMRs and hypoDMRs, respectively. **d** Gene relation annotation of cohort 1 hyperDMRs and hypoDMRs, respectively. The percentage of hyperDMRs or hypoDMRs, respectively, associated with a given annotation is shown on top of bars. Elements in (**a**) were created in BioRender: Sørensen, K. (2025) https://BioRender.com/khrfkru.

For cfDNA methylome profiling in mCRPC patients, we performed cfMeDIP-seq on plasma samples from 27 mCRPC patients and 10 NPCC men in cohort 1 (Fig. 1a). Clinical characteristics are summarised in Table 1. Plasma ctDNA fractions, as estimated using ichorCNA, ranged from 22.3% to 72.4% in mCRPC patients, while no ctDNA was detected in NPCCs (Fig. S1A and Table S3).

In cohort 1, we identified 38,841 differentially methylated genomic regions (DMRs) in cfDNA from mCRPC patients compared to NPCCs. Of these, 20,654 regions were hypermethylated (hyperDMR) and 18,187 were hypomethylated (hypoDMR) in mCRPC (Fig. 1b and Table S4). DMRs were widely distributed across the human genome (Fig. S1B). The majority of hyperDMRs mapped to CpG-rich genomic elements (CpG islands and shores, 12,348/20,654; Fig. 1c) and/or to genic elements (15,305/20,654; Fig. 1d), while most hypoDMRs mapped to CpG-poor shelves and open sea regions (17,233/18,187; Fig. 1c) and less than half to genic elements (6802/18,187; Fig. 1d). These findings align with the known PC methylome landscape, which is characterised by aberrant hypermethylation of CpG rich elements as well as overall global loss of DNA methylation, as identified in prior tumour tissue-based studies [18–20]. Moreover, several hyperDMRs identified in our analysis were associated to genes previously described as common targets for aberrant hypermethylation in PC tumours (e.g. *GSTP1*, *RASSF1*, *CCDC181*, and *HAPLN3* [20–23]*;* Table S4), further supporting the validity of our cfDNA-based findings.

### Development of mCRPC-specific cfDNA methylation signature; cfMeCaP

**Fig. 2:**
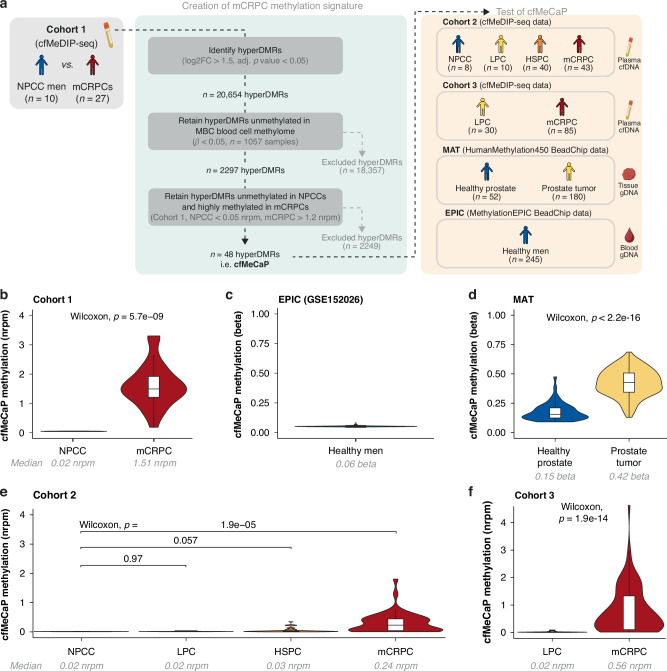
Creating a mCRPC-specific methylation signature. **a** Schematic overview of the creation of cfMeCaP. First, cohort 1 hyperDMRs were filtered against a large reference methylome of blood cell samples (*n* = 1057) to retain only regions that were also unmethylated in blood cells. Next, based on cohort 1 methylation levels, hyperDMRs that were highly methylated in mCRPC patients (>1.2 nrpm) while unmethylated in NPCCs (<0.05 nrpm) were retained. This filtration identified 48 genomic regions (the cfMeCaP signature). cfMeCaP methylation levels were tested in 4 different datasets to evaluate the signature’s association to PC. **b** Mean cfMeCaP methylation of cohort 1 (cfMeDIP-seq data). **c** Mean cfMeCaP methylation of 245 healthy men from GSE152026 (EPIC data). **d** Mean cfMeCaP methylation of 52 healthy prostate samples and 180 primary prostate tumour samples from the Marmal-aid database (Marmal-aid tissue; MAT, 450 K data). **e** Mean cfMeCaP methylation of cohort 2 (cfMeDIP-seq data). **f** Mean cfMeCaP methylation of cohort 3 (cfMeDIP-seq data). Nrpm= Normalised reads per million, gDNA genomic DNA. Elements in (**a**) were created in BioRender: Sørensen, K. (2025) https://BioRender.com/l90k0wj.

Next, based on the 20,654 hyperDMRs identified in mCRPC patients in cohort 1, we sought to establish a signature for detection of PC-associated methylated ctDNA (me-ctDNA) in plasma. To improve specificity for PC, we employed two stringent filtration steps (Fig. 2a; see also ‘Materials and methods’). First, we limited our analysis to hyperDMRs that were unmethylated in a large set of blood cell samples from cancer-free men, thereby excluding a total of 18,357 hyperDMRs. Second, we selected hyperDMRs that were unmethylated in cohort 1 NPCCs while highly methylated in cohort 1 mCRPC, thereby excluding  an additional 2249 hyperDMRs (Fig. 2a). This identified 48 mCRPC-associated hyperDMRs, distributed across the whole genome (Fig. S2A and Table S5). We refer to this novel genome-wide methylation signature as the ‘**c**ell-**f**ree DNA **Me**thylation signature for **Ca**ncer of the **P**rostate’ (cfMeCaP; Fig. 2a).

As expected, in cohort 1, cfMeCaP showed significantly (*p* < 0.001) higher methylation levels in mCRPC patients relative to NPCCs (Fig. 2b). In further support of its PC-specificity, cfMeCaP methylation levels were extremely low (median *β* = 0.06) in blood cell samples from an external cohort of 245 healthy men (EPIC dataset, Fig. 2c), while they were significantly higher in an external set of 180 prostate tumour tissue (median *β* = 0.42) compared to 52 healthy prostate tissue samples (median *β* = 0.15) (*p* < 0.001; MAT dataset, Fig. 2d).

### cfMeCaP is consistently methylated across PC cohorts

For independent validation, we first tested cfMeCaP in cohort 2 (Fig. 2a and Table 1), comprising plasma samples from 43 mCRPC patients and 8 men without cancer (NPCCs), alongside 40 HSPC and 10 LPC patients to investigate if cfMeCaP signals were detectable in earlier-stage PC. In cohort 2, we observed significantly higher cfMeCaP levels in mCRPC patients (median: 0.24 nrpm) compared to NPCCs (median: 0.02 nrpm; *p* < 0.001; Fig. 2e). Moreover, no significant difference in cfMeCaP methylation levels was observed between LPC (median 0.02 nrpm), HSPC (median: 0.03 nrpm), and NPCC men (median 0.02 nrpm) in this cohort (*p* > 0.05; Fig. 2e). Consistent with this, in an external validation cohort (cohort 3, Table S1) of plasma samples from 85 mCRPC patients and 30 LPC patients, we observed significantly higher cfMeCaP levels in mCRPC patients (median: 0.56 nrpm) compared to LPC patients (median: 0.02 nrpm; *p* < 0.001; Fig. 2f). Together, these results confirmed the consistency of cfMeCaP across multiple PC cohorts.

### cfMeCaP enables efficient detection of plasma me-ctDNA

**Fig. 3:**
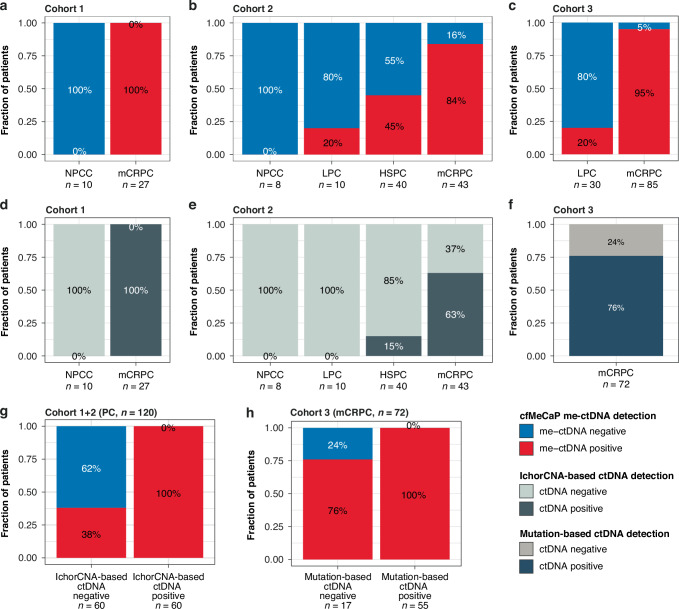
Detection of (me-)ctDNA in PC plasma. **a** Detection of methylated ctDNA (me-ctDNA) using the cfMeCaP signature in cohort 1. **b** cfMeCaP me-ctDNA detection in cohort 2 (internal validation). **c** cfMeCaP me-ctDNA detection in cohort 3 (external validation). **d** Detection of ctDNA, as estimated from copy number profiles using ichorCNA, in cohort 1. **e** IchorCNA copy number-based ctDNA detection in cohort 2. **f** Detection of ctDNA, as estimated from mutation calls from targeted sequencing data, in a subset of cohort 3 (72 mCRPC patients). **g** cfMeCaP me-ctDNA detection stratified by IchorCNA copy number-based ctDNA detection in patients from cohort 1 and 2 (*n* = 120). **h** cfMeCaP me-ctDNA detection stratified by mutation-based ctDNA detection in a subset of cohort 3 mCRPC patients (*n* = 72).

To further explore the potential of cfMeCaP as a tool to detect me-ctDNA in plasma from PC patients, we defined a cutoff (0.0289 nrpm) for me-ctDNA detection based on cfMeCaP methylation levels of NPCCs in cohort 1 (see ‘Materials and methods’) and used it to classify patients as me-ctDNA positive or negative. Accordingly, cfMeCaP detected me-ctDNA in all mCRPC patients in cohort 1, while none of the NPCC men were me-ctDNA positive (Fig. 3a). Testing this cutoff, cfMeCaP detected me-ctDNA in 84% of mCRPC patients in cohort 2 (Fig. 3b), whereas 95% of mCRPC patients were me-ctDNA positive in cohort 3 (Fig. 3c). Furthermore, cfMeCaP was able to detect me-ctDNA in 20% of LPC and 45% of HSPC patients in cohort 2 (Fig. 3b), as well as 20% of LPC patients in cohort 3 (Fig. 3c).

To compare cfMeCaP me-ctDNA detection with routine clinical markers, we assessed its relationship with PSA and metastatic characteristics for PC patients in cohorts 1–3. We observed no differences in serum PSA levels between me-ctDNA positive and negative PC patients in any of the cohorts (*p* > 0.05, Fig. S3A–C). Among mCRPC patients, where all individuals had metastatic disease, me-ctDNA detection appeared to vary by metastatic site, with the highest detection rates observed in those with visceral involvement (Fig. S3D–F). In HSPC, me-ctDNA positivity was enriched in patients with M1 compared to M0 disease (*p* < 0.001, McNemar’s tests, Fig. S3G).

### cfMeCaP is superior to genomics-based ctDNA detection strategies

Next, we explored the performance of cfMeCaP in relation to a copy number-based (i.e. ichorCNA analysis of LPWGS data) and a mutation-based (i.e. deep targeted sequencing of 72 mCRPC driver genes) ctDNA detection strategy. While the copy number-based strategy, performed on cohort 1 and cohort 2 patients, achieved 100% sensitivity in cohort 1 (Fig. 3d), this strategy was inferior to cfMeCaP in cohort 2, with none of the LPC patients, and only 15% of the HSPC and 63% of the mCRPC patients in this cohort being identified as ctDNA positive (Fig. 3e and Table S6). Similarly, a mutation-based detection strategy, performed on a subset of mCRPC patients in cohort 3 (*n* = 72), detected ctDNA in only 76% of the mCRPC patients (Fig. 3f and Table S6). Furthermore, cfMeCaP detected me-ctDNA in all PC samples classified as ctDNA positive by either the copy number (cohort 1 + 2, *n* = 120) or the mutation-based (cohort 3, *n* = 72) strategies, and an additional 38% and 76% of samples classified as ctDNA negative by the copy number and mutation-based strategies, respectively (Fig. 3g, h). Together, our results support the superior sensitivity of the cfMeCaP-based me-ctDNA detection strategy.

### Longitudinal cfMeCaP profiling identifies early treatment failure

**Fig. 4:**
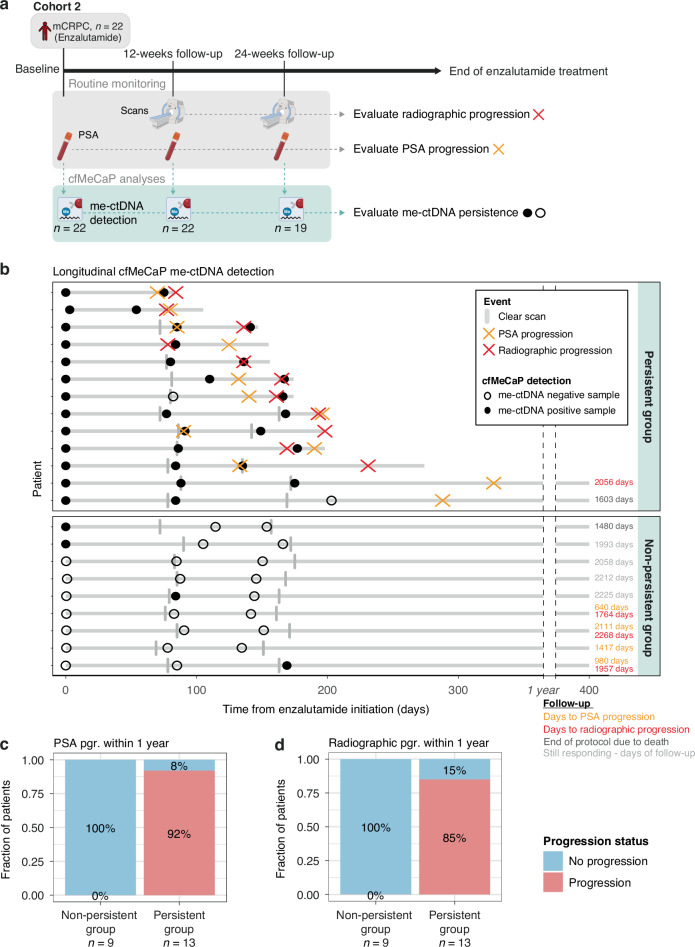
Longitudinal analyses of plasma cfDNA. **a** Schematic overview of the longitudinal plasma sample collection in a subset of cohort 2 mCRPC patients receiving enzalutamide as first-line mCRPC treatment (*n* = 22). Patients were monitored for disease progression at 12 weeks and 24 weeks after treatment initiation using routine PSA measurements and scans. Plasma samples were collected at baseline, at the 12-week follow-up scan, and at the 24-week follow-up scan, and subjected to whole methylome profiling followed by cfMeCaP analyses. **b** cfMeCaP me-ctDNA detection analyses in cohort 2 longitudinal samples. Based on the longitudinal me-ctDNA detection status, patients were split into two me-ctDNA dynamics groups: the Non-persistent group (*n* = 9) and the Persistent group (*n* = 13). Days to follow-up events outside the range of the figure are indicated by colour coding: Yellow: Days to PSA progression, Red: Days to radiographic progression, Black: End of protocol due to death, Grey: Patient still responding – days to last follow-up. **c** cfMeCaP me-ctDNA dynamics groups stratified by the development of PSA progression within 1 year from enzalutamide initiation. **d** cfMeCaP me-ctDNA dynamics groups stratified by the development of radiographic progression within 1 year from enzalutamide initiation. Elements in (**a**) were created in BioRender: Sørensen, K. (2025) https://BioRender.com/3fq8x90.

To explore the potential of cfMeCaP in predicting early treatment failure in mCRPC patients, we combined cfMeCaP me-ctDNA detection with longitudinal plasma sampling at weeks 12 and 24, using 22 mCRPC patients from cohort 2 (Fig. 4a). Response to treatment was monitored using routine PSA measurements and scans every 12 weeks. In this cohort, all patients with detectable me-ctDNA at baseline and in at least one follow-up sample (i.e. Persistent group, *n* = 13) had either primary treatment resistance or had PSA and/or radiographic progression within 1 year of treatment initiation (Fig. 4b–d). In contrast, none of the patients with undetectable me-ctDNA at baseline or undetectable in both follow-up samples (i.e. Non-persistent group, *n* = 9) experienced PSA or radiographic progression within 1 year of treatment initiation (Fig. 4b–d). On median, patients in the Persistent group had PSA-PFS and radiographic PFS (rPFS) of 4.4 months and 5.4 months, respectively, compared to 65.5 months and 67.5 months for patients in the Non-persistent group. These results indicate that longitudinal me-ctDNA detection during 1st-line enzalutamide treatment is associated with early treatment failure.

### cfMeCaP methylation at mCRPC baseline is associated with poor outcome

**Fig. 5:**
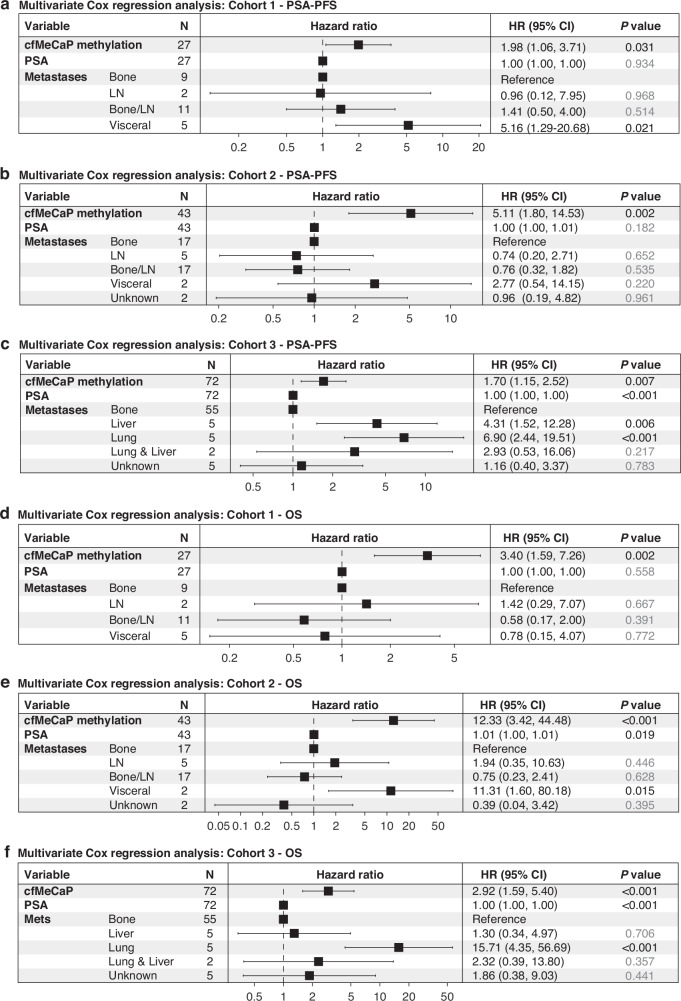
Multivariate analysis of cfMeCaP methylation at mCRPC baseline. **a** Multivariate cox-regression analysis in cohort 1 using PSA-PFS as endpoint. **b** Multivariate cox-regression analysis in cohort 2 using PSA-PFS as endpoint. **c** Multivariate cox-regression analysis in cohort 3 using PSA-PFS as endpoint. **d** Multivariate cox-regression analysis in cohort 1 using OS as endpoint. **e** Multivariate cox-regression analysis in cohort 2 using OS as endpoint. **f** Multivariate cox-regression analysis in cohort 3 using OS as endpoint. All parameters except “Metastases” are analysed as continuous variables. Grey *p*-values indicate non-significance (*p* > 0.05). HR hazard ratio, LN lymph node metastases.

Finally, we explored the prognostic potential of cfMeCaP at mCRPC baseline. Given that most patients had detectable me-ctDNA at mCRPC baseline (100%, 84% and 95% in cohorts 1, 2, and 3, respectively), using me-ctDNA detectability for prognostic stratification would result in highly unbalanced groups, limiting statistical power (Fig. S4). Therefore, we instead focused on the cfMeCaP methylation levels. In cohort 1, high cfMeCaP methylation was associated with significantly shorter time to PSA-progression on first-line ARSI treatment in both Kaplan–Meier (*p* = 0.014; Fig. S5A) and univariate cox regression (*p* = 0.027; Fig. S6A) analyses. This association was validated in both cohort 2 (*p* < 0.015; Figs. S5B and S6B) and cohort 3 mCRPC patients (*p* < 0.002; Figs. S5C and S6C). In line with these findings, high cfMeCaP methylation levels were associated with significantly shorter OS for mCRPC patients in cohort 1 (*p* < 0.009; Figs. S5D and S6D), which was validated in cohort 2 (*p* < 0.002; Figs. S5E and S6E) and cohort 3 (*p* < 0.001; Figs. S5F and S6F). Furthermore, in multivariate Cox regression analyses, high cfMeCaP methylation remained a significant adverse predictor of PSA-PFS and OS, independent of previously described prognostic clinical features (i.e. serum PSA level and localisation of the metastases) in all three mCRPC cohorts (*p* < 0.05; Fig. 5a–f). Taken together, our results emphasise the promising clinical potential of using cfDNA-derived multi-marker methylation signatures for mCRPC prognostication.

## Discussion

In the present study, we used methylome profiling of plasma cfDNA from patients with mCRPC and men without cancer to show that clinically relevant genome-wide methylation markers can be identified in cfDNA from liquid biopsies. As a significant proportion of cfDNA arise from blood cells [24], tumour-specific cell-free methylation signals may become diluted by the methylation signals from blood cells, as evident from e.g. the low sensitivity of certain methylation-based classifiers in predicting different cancer types [25–27]. One approach to increase tumour specificity of methylation signals is to exclude blood cell-derived methylation signals from cfDNA methylomes using parallel whole-methylome sequencing of matched blood cell DNA [28]. Although this approach may effectively remove patient-specific blood cell methylation signals, it comes with a substantial increase in analytical costs. Instead, in our analysis, we generated an in-silico reference methylome of blood cell DNA from a large cohort of cancer-free men, using publicly available methylation data, and subsequently restricted our analyses to regions that are unmethylated in blood cells. To reduce the potential influence of age-associated methylation changes in blood cells of older adult men, NPCCs in this study were selected to match the age range of the patients, and the blood cell reference methylome was derived predominantly from adult donors (median age 60 years).

The genome-wide methylation signature (cfMeCaP) established in this study consisted of 48 genomic regions that were significantly hypermethylated in plasma from mCRPC patients compared to NPCCs. While plasma cfMeCaP methylation in LPCs or HSPCs did not significantly differ from NPCCs, we showed that cfMeCaP was hypermethylated in primary PC tumours compared to healthy prostate tissue, supporting the biological validity of the signature. Consistent with this, multiple cfMeCaP regions were associated with gene families previously described as hypermethylated in primary PC tumours compared to healthy prostate tissue, including e.g. the LIM homeobox gene family (*LHX6*, *LHX9* [29–31]), the ANTP homeobox gene family (*HOXD1* [29, 31]), and the Oligodendrocyte Transcription Factor gene family (*OLIG2*, *OLIG3* [29, 31, 32]). Additionally, one cfMeCaP region (cfMeCaP region 3) was annotated to the *CCDC181* promoter, a region previously reported as part of a three-gene prognostic methylation signature in PC [20].

The cfMeCaP signature was capable of highly sensitive detection of me-ctDNA in mCRPC patients in all three cohorts (84–100% sensitivity). This is in accordance with previous studies, which in general have reported high sensitivity for plasma ctDNA detection as well as high ctDNA% in mCRPC patients, detected using different strategies such as mutations [33, 34] or copy-number variation [35, 36]. We also compared the performance of cfMeCaP to alternative ctDNA detection approaches. We found that cfMeCaP outperformed ichorCNA, a method based on copy-number variation estimated from low-coverage WGS data. While differences in sequencing depth (ichorCNA-based: ~0.5x, cfMeCaP-based: ~1.5x) may contribute to discrepancies in the performance of these strategies, the cfMeCaP strategy also outperformed a mutation-based ctDNA detection strategy, in which the ctDNA% was estimated from targeted sequencing data with a median sequencing depth of 688x [10]. Together, our findings highlight that a multi-methylation marker signature shows great potential in tumour-naïve ctDNA detection in mCRPC.

Using our cfMeCaP methylation signature, the majority of mCRPC patients had detectable levels of me-ctDNA at the time of mCRPC diagnosis. When assessed for its prognostic potential, we found high cfMeCaP methylation levels at mCRPC baseline to be an adverse predictor of both PSA-PFS and OS, independent of established clinical parameters such as serum PSA levels or the localisation of metastasis. Our results confirm and expand on previous studies that have also reported on the prognostic biomarker potential of ctDNA methylation in mCRPC [9, 37–39] as well as other cancer types, including head and neck squamous cell carcinoma (HNSCC) [28] and hepatocellular carcinoma [40]. Several studies have reported genomics-based ctDNA% as an adverse predictor of PSA-PFS and OS in mCRPC [33–35], suggesting that ctDNA methylation may primarily reflect plasma ctDNA levels. However, in our analysis, high cfMeCaP methylation remained a significant adverse predictor of both PSA-PFS and OS, independent of the ichorCNA-based and mutation-based ctDNA% in cohort 1 and 3 mCRPC patients, respectively (Fig. S7A–F). In accordance with this, a previous study reported a composite methylation score of 5 genomic regions to hold prognostic potential independent of ctDNA abundance in HNSCC patients [28]. These findings suggest that cfDNA methylation analysis may provide a refined prognostication of mCRPC patients, possibly due to its improved analytical sensitivity. To support clinical implementation, larger independent cohorts are needed to validate cfMeCaP as a prognostic biomarker for mCRPC, either as a continuous measure or to define cohort- or context-specific cutoffs if dichotomisation is desired.

Moreover, we showed that longitudinal testing of me-ctDNA during the initial months of mCRPC treatment could identify patients with rapid disease progression, for whom treatment intensification might provide additional clinical benefits. In line with these findings, a previous study showed significantly shorter rPFS in mCRPC patients with detectable ctDNA at baseline and after 9 weeks of treatment (enzalutamide w/o atezolizumab), compared to patients with undetectable ctDNA at both time points [34]. Extensive research efforts are currently directed towards demonstrating the clinical utility of ctDNA-guided monitoring of therapy response in multiple cancer types. For example in patients with advanced non-small-cell lung cancer undergoing systemic therapy, ctDNA-based molecular progression was detectable before radiographic progression [41]. The study concluded that a third of all therapy cycles were likely ineffective as they were administered after molecular progression. Such a conclusion might also have important significance for mCRPC treatment, as 20-40% of mCRPC patients are known to harbour primary ARSI resistance and are less likely to benefit from ARSI, the current mainstay for mCRPC treatment [42–44]. Noteworthily, we further showed that a me-ctDNA analysis at baseline and 12 + 24 weeks was able to identify patients who did not progress within 12 months of treatment initiation. For these patients, intensive monitoring of the disease might not be necessary, enabling healthcare resources to be reallocated where they are needed the most.

Although the cfMeCaP signature was developed entirely in the mCRPC setting, it enabled me-ctDNA detection in one in five LPC patients and approximately every other HSPC patient. While low ctDNA abundance is generally characteristic of earlier stages of PC, previous studies have associated detectable ctDNA with rapid disease progression in both LPC and HSPC [23, 45, 46]. Given the small sample size and limited clinical information of LPC/HSPC patients in our study, larger cohorts with longitudinal follow-up are now warranted to determine the prognostic utility of the cfMeCaP signature in early-stage and treatment-naïve PC.

Our study is, however, not without limitations. First, due to the inclusion of only a few NPCCs, available 450 K data were used to exclude methylation signals from normal blood. This restricted our signature to regions that are covered by the 450 K array. Second, by focusing the signature development on mCRPC patients, methylation signals from early-stage PC could potentially be missed, in turn limiting the sensitivity of cfMeCaP in these early stages. Third, since cfMeCaP relies on plasma ctDNA, tumours that do not shed detectable levels of ctDNA might be missed by this method. Fourth, additional prognostic clinical markers, such as LDH or neutrophil-to-lymphocyte ratio, were not available across all mCRPC cohorts and therefore could not be included in the survival analyses. Finally, while the longitudinal me-ctDNA dynamics show promising clinical potential, larger studies are needed to further clarify the association between me-ctDNA dynamics and response to therapy in patients with mCRPC.

## Conclusions

In conclusion, this study established a tumour-naïve signature (cfMeCaP) for the detection of methylated ctDNA in PC. Compared to existing copy number/mutation-based methods, our cfMeCaP signature showed superior sensitivity in the detection of cancer across different stages of PC. High cfMeCaP at mCRPC baseline was significantly associated with poor PSA-PFS and OS, and cfMeCaP-based me-ctDNA dynamics across time-points proved to be an effective indicator of early treatment failure, strongly encouraging further clinical validation of these results.

## Supplementary information

Supplementary Files

Supplementary Figure 1

Supplementary Figure 2

Supplementary Figure 3

Supplementary Figure 4

Supplementary Figure 5

Supplementary Figure 6

Supplementary Figure 7

Supplementary Table 1

Supplementary Table 2

Supplementary Table 3

Supplementary Table 4

Supplementary Table 5

Supplementary Table 6

## Data Availability

The data generated in this study are available through restricted access from GenomeDK (https://genome.au.dk/) under the accession number GDK000020 (https://genome.au.dk/library/GDK000020). All other data are available within the article or its Supplementary Materials. External datasets are available from their respective repositories, as described in the ‘Materials and methods’.
