# Supplementary material for: Genome-wide methylome profiling of cell-free DNA enables prognostication of patients with castration-resistant prostate cancer
Source: Br J Cancer. 2026 Apr 10;135(1):127–38. doi: 10.1038/s41416-026-03432-y (PMC13269899; doi:10.1038/s41416-026-03432-y)
Supplement: Supplementary file 1 — Supplementary Files [file 41416_2026_3432_MOESM1_ESM.pdf]

Supplementary files

Supplementary table 1: Cohort 3 clinical information

Supplementary table 2: Overview of Marmal-aid samples

Supplementary table 3: Library information

Cohort 1 and 2 cfMeDIP-seq library information.

Recovery(%)_meth: Recovery of methylated DNA, Recovery(%)_Unmeth: recovery of unmethylated DNA, Specificity: specificity of immunoprecipitation, Satur_TruCor: true saturation estimated using the MEDIPS R package, Satur_EstCor: estimated saturation (MEDIPS R package), CpG_Enrich_relH: Relative CpG enrichment (MEDIPS R package), CpG_Enrich_GoGe: observed/expected ratio of CpGs (MEDIPS R package).

Supplementary table 4: Cohort 1 DMRs

Nrpm: normalized reads per million, Log2FC: Log_2 fold change.

Supplementary table 5: cfMeCaP regions

Log2FC: Log_2 fold change between NPCC and mCRPC methylation in cohort 1, Adj. P-value: false discovery rate-corrected P-value.

Supplementary table 6: Performance of (me-)ctDNA detection strategies

Supplementary figure 1: Methylome profiling of plasma cell-free DNA

A. Upper panel: ctDNA fractions (ctDNA%), as estimated from copy number profiles using ichorCNA in NPCCs and mCRPC patients from cohort 1. Lower panel: plasma cfDNA concentrations (ng/mL) of NPCCs and mCRPC patients in cohort 1.
B. The distribution of hyperDMRs and hypoDMRs identified in cohort 1. Red lines represent hyperDMRs, blue lines represent hypoDMRs. The thickness of lines is not normalized according to chromosome lengths.

Supplementary figure 2: cfMeCaP regions

A. Chromosomal distribution of the 48 genomic regions of the cfMeCaP signature and mean methylation levels per region for cohort 1 NPCCs and mCRPC patients respectively.

Nrpm: normalized reads per million.

Supplementary figure 3: Comparison of cfMeCaP me-ctDNA detection and routine clinical parameters.

A. Serum PSA levels of cfMeCaP me-ctDNA negative vs positive men in cohort 1.
B. Serum PSA levels of cfMeCaP me-ctDNA negative vs positive men in cohort 2.
C. Serum PSA levels of cfMeCaP me-ctDNA negative vs positive men in cohort 3.
D. cfMeCaP me-ctDNA detection by metastatic site of mCRPC patients in cohort 1.

E. cfMeCaP me-ctDNA detection by metastatic site of mCRPC patients in cohort 2.
F. cfMeCaP me-ctDNA detection by metastatic site of mCRPC patients in cohort 3.
G. cfMeCaP me-ctDNA detection by metastasis status of HSPC patients in cohort 2.

NPCC: Non-prostate cancer controls, mCRPC: metastatic castration-resistant prostate cancer, n.d.: no data, LPC: localized prostate cancer, HSPC: hormone-sensitive prostate cancer, LN: lymph node, Bone&LN: Bone and lymph node

Supplementary figure 4: Prognostic potential of plasma cfMeCaP me-ctDNA status at mCRPC baseline.

A. Kaplan-Meier analyses of PSA-PFS stratified by cfMeCaP me-ctDNA detection in cohort 2 mCRPC patients.
B. Kaplan-Meier analyses of PSA-PFS stratified by cfMeCaP me-ctDNA detection in subset of cohort 3 mCRPC patients (n=72).

In cohort 1, all patients were me-ctDNA positive at mCRPC baseline, and therefore this analysis could not be performed. P-values for Kaplan Meier survival analyses were calculated using log-rank test.

Supplementary figure 5. Prognostic potential of plasma cfMeCaP methylation levels at mCRPC baseline.

A. Kaplan Meier survival analysis of cfMeCaP methylation (</≥1.513nrpm) in cohort 1 mCRPC patients using PSA-PFS as endpoint.
B. Kaplan Meier survival analysis of cfMeCaP methylation (</≥0.237nrpm) in cohort 2 mCRPC patients using PSA-PFS as endpoint.
C. Kaplan Meier survival analysis of cfMeCaP methylation (</≥0.455nrpm) in a subset of cohort 3 mCRPC patients (n=72) using PSA-PFS as endpoint.
D. Kaplan Meier survival analysis of cfMeCaP methylation (</≥1.513nrpm) in cohort 1 mCRPC patients using OS as endpoint.
E. Kaplan Meier survival analysis of cfMeCaP methylation (</≥0.237nrpm) in cohort 2 mCRPC patients using OS as endpoint.
F. Kaplan Meier survival analysis of cfMeCaP methylation (</≥0.455nrpm) in a subset of cohort 3 mCRPC patients (n=72) using OS as endpoint.

P-values for Kaplan Meier survival analyses were calculated using log-rank test.

Supplementary figure 6: The prognostic potential of cfMeCaP methylation at mCRPC baseline

A. Univariate cox-regression analysis in cohort 1 using PSA-PFS as endpoint.
B. Univariate cox-regression analysis in cohort 2 using PSA-PFS as endpoint.
C. Univariate cox-regression analysis in cohort 3 using PSA-PFS as endpoint.
D. Univariate cox-regression analysis in cohort 1 using OS as endpoint.
E. Univariate cox-regression analysis in cohort 2 using OS as endpoint.
F. Univariate cox-regression analysis in cohort 3 using OS as endpoint.

All parameters except “Metastatic volume” are analyzed as continuous variables. P-values are adjusted for multiple testing (Benjamini & Hochberg; BH). Grey p-values indicate non-significance (p>0.05).

CN-based: copy number-based, HR: Hazard ratio, LN: Lymph node metastases

Supplementary figure 7. Prognostic potential of cfMeCaP methylation independent of the ctDNA% at mCRPC baseline

A. Multivariate cox-regression analysis in cohort 1 using PSA-PFS as endpoint.
B. Multivariate cox-regression analysis in cohort 2 using PSA-PFS as endpoint.
C. Multivariate cox-regression analysis in cohort 3 using PSA-PFS as endpoint.
D. Multivariate cox-regression analysis in cohort 1 using OS as endpoint.
E. Multivariate cox-regression analysis in cohort 2 using OS as endpoint.
F. Multivariate cox-regression analysis in cohort 3 using OS as endpoint.

Grey p-values indicate non-significance ($p>0.05$).

ctDNA% (CN): Copy number-based ctDNA%, ctDNA% (mut): mutation-based ctDNA%, HR: Hazard ratio.

Supplementary methods

Blood sample processing

Blood samples from patients recruited to AUH and VH were collected in BS Vacutainer K_2 EDTA tubes and processed within 2 hours. Plasma was separated from cellular components by centrifugation (3000g for 10 minutes at 20°C) and stored in cryo tubes (TPP) at -80°C until extraction of cfDNA.

For cfDNA extraction, plasma samples were equilibrated to room temperature and centrifuged at 3000g for 10 minutes at 20°C. cfDNA from 2-4mL plasma was extracted using the QIAamp Circulating Nucleic acid kit (Qiagen) according to the manufacturer's instructions, either using a Qiasymphony robot (Qiagen) or manually using the QIAvac 24 Plus system (Qiagen). Extracted cfDNA was eluted in LoBind tubes (Eppendorf AB) and stored at -80°C until further use.

cfDNA concentration and quality control was evaluated by multiplex droplet digital PCR (ddPCR), as described previously(1), using the QX200 AutoDG ddPCR System (Bio-Rad). cfDNA was quantified using two assays targeting regions on chromosome 3 and 7, respectively, that rarely show copy number alterations in cancer, including PC (1). cfDNA concentrations were determined as the average of the two assays. For quality control, two additional ddPCR assays were run to assess the cfDNA extraction efficacy and potential contamination of DNA from lysed peripheral blood mononuclear cells (PBMC). To evaluate the cfDNA extraction efficacy, a fixed amount of soybean-specific CCP1 DNA fragments was spiked in each sample prior to extraction. Using a ddPCR assay targeting the spiked in CCP1 fragments, the extraction efficacy was calculated as the percentage of recovered spiked in CCP1 DNA fragments in each sample (1). PBMC contamination was assessed using a ddPCR assay targeting a B lymphocyte-specific locus.

cfMeDIP-seq library preparation

Cell-free methylated DNA immunoprecipitation sequencing (cfMeDIP-seq) libraries were prepared in accordance with the published protocol by Shen et al (2), with slight adjustments. For each sample, up to 100 ng cfDNA (median=30.3ng) was used as input for library preparation using the KAPA HyperPrep kit (KAPA Biosystems) according to manufacturer's instructions, except for only using half volumes of all reagents. In brief, input cfDNA was subjected to end-repair and A-tailing, before ligation of xGen CS-adapters – Tech Access (IDT-DNA) and clean-up using 1.4x AMPure XP Beads (Beckman Coulter). Following library preparation, the total amount of input DNA

in each sample was equalized to 100 ng by the addition of lambda filler DNA, containing a 1:1 ratio mixture of unmethylated and in vitro methylated lambda phage DNA amplicons (2).

Lambda filler DNA-combined libraries were then subjected to MeDIP using the MagMeDIP qPCR kit (Diagenode) according to the published cfMeDIP-seq protocol (2). To allow for quality control after MeDIP, 0.3 ng of methylated and unmethylated *Arabidopsis thaliana* control DNA (DNA methylation control package, Diagenode), respectively, were initially added to each library. Next, libraries were denatured into single-stranded DNA to allow for antibody capture of methylated fragments. Each library was then partitioned into two aliquots: 7.9 µL was collected as input control (IC sample) and stored at 4°C, and 79 µL to be subjected to immunoprecipitation (IP sample). For the immunoprecipitation, IP samples were incubated with anti-5mC monoclonal antibodies and magnetic beads (MagMeDIP qPCR kit, Diagenode) for 17 hours overnight rotation at 4 °C. Following the incubation, IP samples were washed and eluted using the MagMeDIP qPCR kit (Diagenode) and lastly, IP and IC samples were purified and eluted in 50 µL buffer C using the IPure kit v2 (Diagenode) according to the published cfMeDIP-seq protocol (2).

To assess the performance of the immunoprecipitation, qPCR reactions (QC1) were set up separately for IP and IC samples to estimate the recovery of methylated and unmethylated *Arabidopsis thaliana* control DNA (primers provided in the DNA methylation control package, Diagenode), respectively, as described previously (2). After the immunoprecipitation, a median of 37.2% (12.8%-106.2%) methylated DNA and 0.13% (0.04%-0.7%) unmethylated DNA was recovered in the samples, yielding a median specificity of the immunoprecipitation of 99.6% (98.3%-99.9%). Moreover, a second qPCR (QC2) was performed to determine the optimal number of cycles for library amplification. Here, reactions were set up to mimic the subsequent library amplification, using KAPA HiFi Hotstart ReadyMix (Roche) and uniquely indexed primer pairs (IDT-DNA) for each sample. Additionally, SYBR Green I Nucleic Acid Gel Stain (Thermo Fischer Scientific) diluted 1:1,000 in molecular-grade water was added to the reaction to allow visualization and quantification of DNA during qPCR. Reactions were run with the following conditions: initial denaturation at 98 °C for 45s; 25 cycles: denaturation at 98 °C for 15 s, annealing at 60 °C for 30 s, and extension at 72 °C for 30 s; followed by a final extension of 72 °C for 1 min.

Finally, each IP and IC sample was amplified using the KAPA HiFi Hotstart ReadyMix (Roche) and uniquely indexed primer pairs (IDT-DNA) based on the number of cycles determined in QC2. Indexed libraries were purified using 1.0x AMPure XP Beads (Beckman Coulter) and quantified using the Qubit dsDNA High Sensitivity assay (Thermo Fischer Scientifics). Fragment lengths were assessed using the Tapestation 4200 system (HSD1000, Agilent Technologies).

Selection of Marmal-aid samples for reference methylome

A total of 14586 samples were retrieved from the Marmal-aid database. Samples were excluded if they originated from female donors, cell lines, unknown tissues, blood cancers, fetal/placental samples, postmortem biopsies, isolated cell types, or treated tissues. This filtration retained a total of 4204 samples, from which blood cell samples (n=1057), prostate tumor samples (n=180), and healthy prostate samples (n=52) were selected for this study. Blood samples were mainly from older adult males (median=60 years, range: 4-100) of different ethnicities (Caucasian, Hispanic, Japanese) and included both samples from healthy donors as well as donors with lifestyle and age-related conditions.

Processing of external cfMeDIP-seq, 450K and EPIC-based data

For the external cfMeDIP-seq data (cohort 3), raw cfMeDIP-seq IP sample fastq files were retrieved and bioinformatically processed following the approach of cohorts 1 and 2. No IC sample data was available for this cohort, hindering copy number profiling and ctDNA detection using ichorCNA. Instead, copy number variation was assessed directly from the IP samples in the QSEA pipeline (*addCNV* parameter set to *MEDIP=TRUE*) and used for normalization. For 72/85 mCRPC patients, ctDNA% estimated based on allele fractions of autosomal somatic mutations from deep targeted sequencing data (median sequencing depth: 688x) was available and included in the analyses instead (3, 4).

For both 450K and EPIC-based data, samples were batch and peak normalized using the ChAMP R package (v2.16.2), and methylation levels of single CpG sites were averaged into 300 bp genomic windows across the genome to match the data structure of processed cfMeDIP-seq methylation data. Mean methylation levels (beta) of the signature were reported.

Statistical data analyses and clinical outcome

For analyses of signature methylation, methylation levels were either used as continuous variables, or patients were dichotomized into two groups (low and high signature methylation) based on the median signature methylation level of the specific cohort. This was also the case for analyses including the ichorCNA-based ctDNA%. PSA progression was defined as follows: if PSA levels decreased after treatment initiation, PSA progression was defined as an increase in PSA >2ng/mL and >25% from PSA nadir. If PSA levels increased after treatment initiation, PSA progression was defined as an increase in PSA >2ng/mL and >25% 12 weeks from treatment initiation. Radiographic progression was defined according to PCWG3 criteria. Overall survival was defined as the time from initiation of first-line mCRPC treatment until death from any cause.

References

1. Thomas R, Lone VS, Rune T, Heidi T, Søren V, Iver N, et al. Analysis of circulating tumour DNA to monitor disease burden following colorectal cancer surgery. Gut. 2016;65(4):625.
2. Shen SY, Burgener JM, Bratman SV, De Carvalho DD. Preparation of cfMeDIP-seq libraries for methylome profiling of plasma cell-free DNA. Nature Protocols. 2019;14(10):2749-80.
3. Chen S, Petricca J, Ye W, Guan J, Zeng Y, Cheng N, et al. The cell-free DNA methylome captures distinctions between localized and metastatic prostate tumors. Nature Communications. 2022;13(1):6467.
4. Annala M, Vandekerkhove G, Khalaf D, Taavitsainen S, Beja K, Warner EW, et al. Circulating Tumor DNA Genomics Correlate with Resistance to Abiraterone and Enzalutamide in Prostate Cancer. Cancer Discovery. 2018;8(4):444-57.
